# Bictegravir/Tenofovir Alafenamide/Emtricitabine: A Real-Life Experience in People Living with HIV (PLWH)

**DOI:** 10.3390/idr15060069

**Published:** 2023-12-11

**Authors:** Anna Gidari, Sara Benedetti, Sara Tordi, Anastasia Zoffoli, Debora Altobelli, Elisabetta Schiaroli, Giuseppe Vittorio De Socio, Daniela Francisci

**Affiliations:** Department of Medicine, Clinic of Infectious Diseases, “Santa Maria della Misericordia” Hospital, University of Perugia, 06132 Perugia, Italy; sara.benedetti89@libero.it (S.B.); sara.tordi@studenti.unipg.it (S.T.); asia.zoffoli@gmail.com (A.Z.); debora.altobelli@gmail.com (D.A.); elisabetta.schiaroli@unipg.it (E.S.); giuseppedesocio@yahoo.it (G.V.D.S.); daniela.francisci@unipg.it (D.F.)

**Keywords:** HIV, bictegravir, integrase inhibitors, real-life, antiretroviral

## Abstract

Background: Bictegravir (BIC), a recently introduced integrase inhibitor, is available in a single tablet regimen with tenofovir alafenamide (TAF) and emtricitabine (FTC) (BIC-STR). This study aimed to describe a real-life experience with BIC-STR. Methods: We retrospectively analyzed the data of people living with HIV (PLWH) on antiretroviral therapy (ART) with BIC-STR followed by the Clinic of Infectious Diseases of Perugia (Perugia, Italy) from September 2019 to February 2023. Results: 270 PLWH were enrolled with a median follow-up time on BIC-STR of 2.2 years (IQR 1.2–2.7). In the overall population, in treatment-experienced (N = 242), in treatment-naïve (N = 28), and in population with age > 60 years old (N = 86), we observed that CD4 cell count improved in absolute number, percentage and CD4/CD8 ratio, under BIC-STR. Patients with viremia < 50 cp/mL increased in all groups. In the overall population, previous ART with TAF and nadir CD4 cell count favored immunological recovery. In the ART-experienced group, time in therapy with BIC-STR was associated with HIV-RNA undetectability. In the older group, previous opportunistic infection and advanced age were associated with lower CD4 count. Conclusions: BIC-STR was demonstrated, in real-life, to be a valid option for a switch, such as initial ART.

## 1. Introduction

The introduction of antiretroviral therapy (ART) has profoundly changed the clinical outcome of people living with HIV (PLWH), turning a once-fatal disease, heavily laden with acquired immunodeficiency syndrome (AIDS)-related mortalities, into a manageable chronic condition. This shift has notably extended survival times and resulted in an older HIV-infected population [1]. The appearance of integrase inhibitors (INIs) has further enhanced the management of HIV infection. The first INI, raltegravir, was approved in 2007 [1]. Bictegravir (BIC) is a second-generation INI marketed under the name Biktarvy^®^ in a fixed-dose single tablet regimen regimen with emtricitabine (FTC) and tenofovir alafenamide (TAF) (BIC-STR) [2]. It has a high genetic barrier, and in vitro studies have tested its efficacy on wild-type and first-generation INI-resistant strains [3]. Some studies have shown non-inferiority to regimens with elvitegravir (EVG)/booster, protease inhibitors (PIs)/booster, or dolutegravir (DTG), in both experienced and naïve patients [4,5,6]. Bictegravir is recommended by European guidelines for the treatment of naïve and experienced HIV-1 infections in adults [2]. However, clinical practice data are still lacking, such as data on long-term viro-immunological efficacy, metabolic modifications, and efficacy on special populations (i.e., older patients).

The objective of our study is to evaluate the efficacy, safety, and durability in a real-life cohort followed at the Clinic of Infectious Diseases of Perugia, Perugia, Italy. We also separately examined treatment-experienced, naïve patients, and the over 60 years-old subgroups, also evaluating the possible conditions that determine a delay in immunological recovery and virological suppression.

## 2. Materials and Methods

### 2.1. Patients and Data Collection

This study aimed to evaluate changes in CD4 count, viral load, and metabolic profile after BIC-STR introduction. Effectiveness, defined as HIV-RNA < 50 copies/mL, was evaluated on the basis of treatment. Furthermore, variables that influence the residual viremia, defined as detectable HIV-RNA (>20 copies/mL), were evaluated. Virological failure was defined as the presence of two consecutive HIV-RNA > 50 copies/mL taken 1–2 months apart [2,3].

Additional secondary endpoints included factors that influence immunological recovery (defined as CD4 count > 500 cells/mm^3^) in the total cohort and old patients (aged over 60 years old).

Volunteer patients with HIV infection followed by the Clinic of Infectious Diseases of Perugia were enrolled in this study. This study was conducted according to the declaration of Helsinki and approved by the local Ethics Committee; all patients had signed a consent to use their anonymized data when admitted to our department.

We retrospectively analyzed the prospectively collected data of patients on ART with BIC-STR from September 2019 to February 2023. We included all adult patients (age ≥ 18 years) with an established diagnosis of HIV infection. The only exclusion criterion was a follow-up of less than six months after BIC-STR introduction. All data were obtained from electronic medical records (Netcare^®^, Healthware, Salerno, SA, Italy). The collected data were analyzed anonymously. 

The following data were obtained from each patient: demographic characteristics (age, sex), risk factors for HIV infection, Center for Diseases Control (CDC) classification, previous opportunistic infections, pre-BIC-STR (regimen and duration), metabolic profile body mass index (BMI), plasma HIV-RNA, CD4 count, and CD4/CD8 ratio.

Durability, in this context, refers to the length of the ART regimen’s effectiveness, assessed by the incidence of ART suspension for any reason.

### 2.2. Statistical Analysis

Descriptive characteristics were summarized using numbers and percentages for categorical variables, mean and standard deviations (SD), or median and interquartile ranges (IQR) for numerical variables, as appropriate.

The cohort was subcategorized into naïve/experienced and young/old (cut-off: 60 years old) patients.

Furthermore, the variables that correlated to a good immunological recovery (CD4 count > 500 cells/mm^3^) were assessed. The variables selected, based on clinical relevance, were age, sex, time on therapy with BIC-STR, previous opportunistic infections, comorbidities two or more, hepatitis C virus (HCV) or hepatitis B virus (HBV) coinfections, nadir CD4 count, and zenith plasma HIV-RNA. 

Univariate analysis was performed by applying Chi-square, Fisher’s exact test, paired-unpaired *t*-test, Wilcoxon signed-rank test, or Mann–Whitney U test as appropriate. In particular, Wilcoxon signed rank and Mann–Whitney U tests were used if the sample distribution was not normal or if variance was unequal. Multivariate logistic regressions were performed, including the variables found significant in univariate analysis (*p* < 0.05). The results of multivariate logistic regression are given as an odds ratio (OR) with 95% CI and *p*-value. The Hosmer–Lemeshow test was performed to verify if the model was correct.

Statistical analyses were performed using Prism GraphPad 8.3 software (Dotmatics, Boston, Massachussets, MA). A *p*-value < 0.05 was considered significant.

## 3. Results

**Figure 1:**
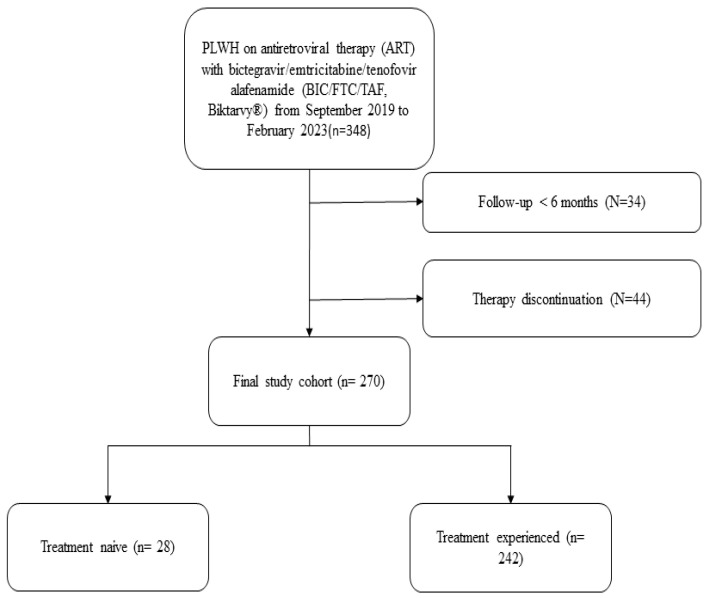
Patient selection flow-chart.

Figure 1 illustrates that 348 patients received BIC-STR treatment at our centre. Of these, 44 patients were not included in this study due to prior discontinuation of the therapy, while 34 were excluded for having a follow-up period of less than six months. The causes of BIC-STR discontinuation were switch to dual therapy (14/44, 31.8%), loss to follow-up (14/44, 31.8%), virological failure (1/44, 2.3%), death (5/44, 11.4%), adverse effects (5/44, 11.4%), drug–drug interaction (2/44, 4.5%), pregnancy planning (1/44, 2.3%), dysphagia/nasogastric tube placement, and the consequent need of crushable tablets (2/44, 4.5%) (Supplementary Figure S1). The most frequent adverse effects were neurological disorders (3/5, 75%) such as sleep disturbances, anxiety, and headache. Other disturbances were myalgia, *prurigo nodularis*, and renal impairment. Among the patients who discontinued BIC-STR, only 20 had a follow-up on the BIC-STR regimen of more than six months. 

The median follow-up time on BIC-STR was 2.2 years (IQR, 1.2–2.7 years).

As shown in Table 1, 270 PLWH were enrolled, of which 81.9% (221/270) were male, with a median age of 54.4 years (IQR 44.6–61.2 years, range 21.4–81.9 years). The majority of the patients were Italian (206/270, 76.3%). More than half of patients presented two or more comorbidities (160/270, 60.1%). Among these, the most represented was dyslipidemia (70/270, 25.9%), followed by hypertension (59/270, 21.9%). Obesity was observed in 29 patients (10.7%) and the population showed an average BMI of 25.7 Kg/m^2^ (IQR 23.2–28.4 Kg/m^2^). Hepatitis C virus infection was observed in 51 patients (19.0%) but only 4 of them had detectable HCV-RNA. However, HBV co-infection occurred in 93 (34.4%) PLWH. Comparing this population with the patients excluded for therapy discontinuation, we found that differences were not significant in demographic characteristics, with the exception of the CD4 nadir that was higher for the excluded cohort and the number of medical examinations per year and higher in the excluded cohort (probably due to the further need of therapy switch).

The cohort showed a mean nadir CD4 count of 190 cells/µL (IQR 50–348 cells/mm^3^) and a HIV-RNA zenith of 163,000 cp/mL (IQR 52,500–642,000 cp/mL). However, 114/270 (42.2%) of patients had a nadir CD4 count < 200 cells/mm^3^, and among these, 72/114 (63.2%) had a prior diagnosis of AIDS. The most representative opportunistic infections were *Pneumocystis jirovecii* pneumonia (21/72, 29.2%), Kaposi sarcoma (14/72, 19.4%), neurotoxoplasmosis (8/72, 11.1%), and oesophageal candidiasis (8/72, 11.1%). Furthermore, 12/72 (16.7%) of patients had two or more opportunistic infections.

At least one genotypic resistance test (before BIC-STR initiation) was performed in 183/270 patients (67.7%). No substitutions affecting INIs’ activity were found in any patients, and 3/183 patients showed M184V mutations. Other mutations were found in 58/183 (31.7%) of patients.

The majority of the patients (242/270, 89.6%) were on ART with a median therapy duration of 10 years (IQR 7–17 years). Before the switch to BIC-STR, our cohort was treated with INIs (187/242 77.3%), non-nucleoside reverse transcriptase inhibitors (NNRTIs) (21/242 8.7%), PIs (36/242, 14.9%), TAF backbone (167/242, 69%), abacavir (ABC) backbone (31/242, 12.8%), and dual-therapy (15/242, 6.2%).

We analyzed how some important values changed after BIC-STR introduction. For this purpose, we separately analyzed treatment-naïve and -experienced patients.

**Figure 2:**
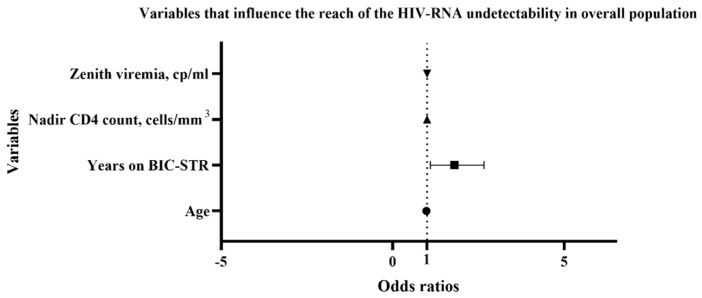
Multiple logistic regression: variables that influence the reach of target undetected viermia. HIV, human immunodeficiency virus; BIC-STR, cp, copies; bictegravir-single tablet regimen.

In the treatment-experienced group (N = 242), the median follow-up time on BIC-STR was 2.5 years (IQR 1.4–2.9 years). In this group, we observed that the CD4 count improved in absolute number, percentage, and CD4/CD8 ratio, after the switch to BIC-STR (see Table 2, *p* < 0.0001). In this group, patients with viremia < 50 cp/mL were 197/242 (81.4%) and became 228 (94.2%) after the switch. The patients with viremia < 50 cp/mL before the switch had a median time of virological suppression of 8.4 years (IQR 5.1–12.8). Considering the HIV-RNA target < 20 cp/mL, 166/242 (69.6%) patients reached this value before the switch, and the percentage significantly increased after the switch (203/242, 83.9%, *p* < 0.0001). Some variables (age, sex, time in therapy with BIC-STR, time in ART pre-switch, previous opportunistic infections, HCV or HBV coinfections, comorbidities more than one, nadir CD4 count, zenith HIV-RNA, previous other ART then BIC-STR) were analyzed to establish which one could be related to the incomplete virological suppression, defined as detectable HIV-RNA (>20 cp/mL). Among these variables, age, time in therapy with BIC-STR, nadir CD4 count, and zenith HIV-RNA were significantly associated with the outcome at the univariate analysis. However, at the multivariate logistic regression, only the time in therapy with BIC-STR was associated with the reach of the HIV-RNA undetectability (OR 1.643, confidence interval, CI, 1.038 to 2.660, Figure 2, Supplementary Table S1).

As shown in Table 2, after the switch to BIC-STR, we found a significant reduction in the total cholesterol and triglycerides, while low-density lipoprotein (LDL) and high-density lipoprotein (HDL) and BMI have not undergone significant variations.

Immuno-virological and metabolic variation after BIC-STR introduction were also analyzed in ART-naïve patients (N = 28) with a median follow-up of 1.8 years (IQR 1.3–2.5). As expected, a significant immune-virological improvement was observed (Table 3). Most of these patients (26/28, 92.8%) showed a good virological response (HIV-RNA < 50 copies/mL) and immunological recovery with a median of CD4 count of 514 cell/mm^3^ (IQR 271.5–697.3). In this group, increases in total cholesterol, LDL, and BMI were observed, while triglycerides and HDL variations were not significant.

**Figure 3:**
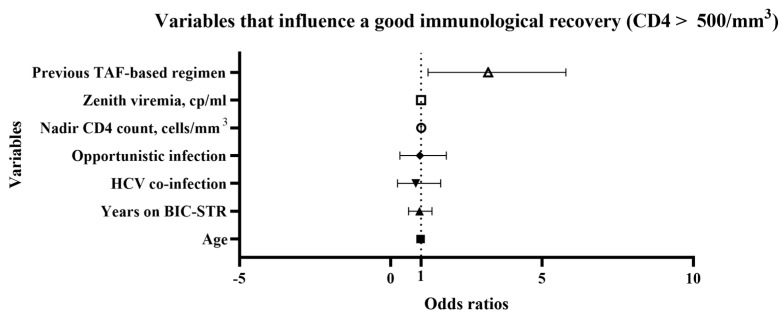
Multiple logistic regression: variables that influence a good immunological recovery, defined as CD4 count > 500 cells/mm^3^, in overall population. TAF, tenofovir alafenamide; cp, copies; HCV, hepatitis C virus; BIC-STR, bictegravir single tablet regimen.

Variables that influence a good immunological recovery, defined as CD4 count > 500 cells/mm^3^, were analyzed in the overall population. Different variables (age, sex, time in therapy with BIC-STR, time in ART pre-switch, previous opportunistic infections, HCV or HBV coinfections, comorbidities more than one, nadir CD4 count, zenith HIV-RNA) were included in the univariate analysis. The variables which resulted significantly associated with the immunological recovery at univariate analysis were subsequently included in multiple logistic regression (previous ART with TAF, nadir CD4 count, zenith HIV-RNA, previous opportunistic infections, HCV coinfection, time in therapy with BIC-STR). Among these variables, only a previous ART with TAF backbone (OR 2.642, CI 1.235 to 5.787) and nadir CD4 cells count (1.006, CI 1.004 to 1.009) were found to influence the immunological recovery (Figure 3, Supplementary Table S2).

Similar analyses were performed for the older group of our population, defined as PLWH aged over 60 years old. This subgroup was composed of 86 patients, with a median age of 64.6 years old (IQR 61.4–68.0). Male sex was prevalent (80/86, 93.0%). Almost one-third had a previous diagnosis of AIDS (26/86, 30.6%). The rate of comorbidities was higher than in the overall population; indeed, 69/86 (80.2%) patients showed more than two comorbidities. The most frequent comorbidities were hypertension (36/86, 41.9%) and dyslipidemia (28/86, 32.6%). The subgroup showed a mean nadir CD4 count of 129 cells/mm^3^ (IQR 48–300 cells/mm^3^) and a HIV-RNA zenith of 246,500 cp/mL (IQR 45,325–852,500 cp/mL). Most of the patients were ART-experienced (81/86, 94.2%) and only five PLWH were naïve. Treatment-experienced patients were on ART for a median period of 144 months (IQR 84–204) before the switch. We analyzed how some important values changed after the BIC-STR introduction in older patients. To this purpose, we excluded treatment-naïve patients. As shown in Table 4, we observed CD4 count, percentage, and CD4/CD8 ratio improvement. The metabolic profile improved with a decrease in total cholesterol and triglycerides. No significant changes in LDL, HDL, and BMI were found.

**Figure 4:**
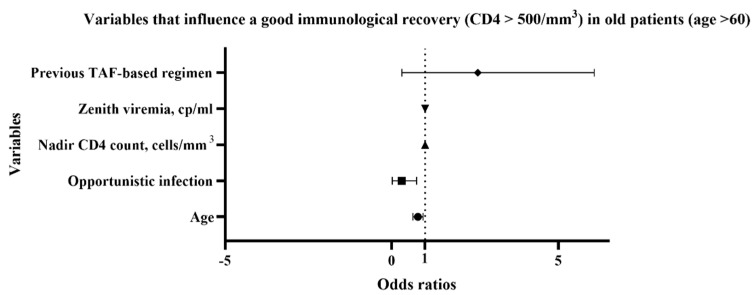
Multiple logistic regression: variables that influence a good immunological recovery (CD4+ T cell count > 500 cells/mm^3^) in patients older than 60 years old. TAF, tenofovir alafenamide; cp, copies.

We also verified which variables influenced a good immunological recovery in older PLWH (age > 60 years old). Previous opportunistic infection (OR 0.1378, CI 0.01913 to 0.7464) and advanced age (OR 0.7879, CI 0.6372 to 0.9386) were associated with a lower CD4 count (<500 cells/mm^3^) (Figure 4, Supplementary Table S3). 

## 4. Discussion

Bictegravir is one of the last introduced INIs, it is available in co-formulation with TAF and FTC (BIC-STR). This combination demonstrated a satisfying efficacy and safety profile. This study aimed to verify these characteristics in a real-life cohort.

In the present study, BIC-STR demonstrated a good safety profile, with a very low incidence of adverse effects (5/348 patients) requiring therapy discontinuation. In all these cases, the events were mild and resolved after the therapy discontinuation. Our results are in line with the available literature: Lagi and colleagues observed a very low rate of discontinuation due to adverse events and self-suspension in patients on BIC-STR therapy. In this study, the first cause of suspension was the simplification to dual therapy [4]. Other studies reported a rate of discontinuation of 13 to 14%, of which 37 to 69% were for toxicity [5,6]. About the second-generation INIs, an important concern is the onset of neuropsychiatric adverse effects (NPAE). Briefly, in this aspect BIC appeared similar to DTG, causing mild to moderate NPAE and, rarely, severe symptoms may lead to treatment discontinuation [7]. In particular, depression reporting was important for both BIC and DTG [8]. In our cohort, among the discontinuation for adverse effect, NPAEs were the most represented, even if the percentage was low. Chen et al. performed a patients-reported outcome study where more than six hundred PLWH, after 48 weeks from the switch to BIC-STR, answered questions about 20 HIV-related symptoms (HIV symptom index). The authors observed that six symptoms were significantly less prevalent, and seven symptoms were significantly less bothersome. Furthermore, participants previously treated with EVG experienced a higher improvement [9].

As previously described in studies documenting the BIC/FTC/TAF non-inferiority compared to other ART regimens, and also in our study, the percentage of PLWH with viral load <50 cp/mL significantly increased after BIC-STR introduction, reaching 94% [5,10,11,12]. Furthermore, our data demonstrated that the percentage of patients reaching the undetectable target (<20 cp/mL) increased with time in BIC-STR therapy. Armenia et al. observed that after 96 weeks of BIC-STR therapy, the percentage of detected viremia is about 5%, without significant difference among patients receiving fully or partially active regimens. They also demonstrated that only patients who had previously experienced INIs failures were at risk of losing virological control under BIC-STR [13]. Other studies confirmed that previous resistance-associated mutations to nucleoside reverse transcriptase inhibitors (such as M184V and K65N/R) are not associated with virological failure after the switch to BIC-STR in virological suppressed PLWH [14,15]. Furthermore, no differences between DTG and BIC regimens have been observed regarding low-level viremia or virological failure onset [15].

An improvement in immunological reconstitution was observed, evident in the absolute CD4 count values, their percentage, and the CD4/CD8 ratio. These data emerged both from the analysis of the overall population and the subgroups (treatment-experienced and naïve) and agreed with the literature: the various studies confirmed the significant increase in CD4 count in terms of absolute number and percentage [5,16]. In the present study, we also analyzed variables that influence a good immunological recovery, defined as CD4 count > 500 cells/mm^3^. In the overall population, variables associated with this outcome were low CD4 nadir count and a previous TAF-based regimen. In particular, low CD4 nadir count predisposed to poor CD4 recovery, while a previous TAF-based regimen promoted a better recovery. This last finding underlines how a strong backbone therapy is still important to reach a good immunological recovery. In the older subgroup (aged over 60 years old), factors associated with CD4 count improvement were age and previous opportunistic infections, underlining the importance of an early diagnosis and treatment, especially in the frailest populations. However, in this subgroup, we still observed an improvement in CD4 count, percentage, and CD4/CD8 ratio, such as in the metabolic profile with a decrease in total cholesterol and triglycerides. Similar findings were described in a recent retrospective Italian study, where a cohort of >55-years-old PLWH were switched to BIC-STR [16]. Another study was performed analyzing a 350-patient cohort with age ≥ 50-years-old who switched to BIC/TAF/FTC, and the authors observed that at week 48, 330 patients maintained viraemia < 50 cp/mL and 20 patients between 50 and 400 cp/mL. Furthermore, many potential drug interactions have been avoided, supporting the efficacy and safety of BIC/TAF/FTC therapy in elderly patients [17].

In the elderly population, a low CD4/CD8 ratio is indicative of a poor prognosis. Although the mechanisms of this effect are unknown, some researchers suggested that the poor CD4 count contributed to a subtle immunodeficiency, while the increased number of potentially dysfunctional CD8 count could create a toxic inflammatory environment [18]. For this reason, the CD4/CD8 ratio could be used as a biomarker for HIV infection. This ratio is very slow to normalize during ART, and the CD8 count value may remain persistently high. For this reason, small and monocentric cohorts have evaluated that a low CD4/CD8 ratio, which determines activation, senescence, and dysfunction of T lymphocytes, is associated with a poor prognosis [19].

Mussini and colleagues analyzed 3236 HIV-infected individuals within the Italian ICONA cohort. During a five-year period of HIV virological suppression, less than one-third (29%; 95% CI, 27–32) of patients had a normalization of CD4/CD8 ratio (≥1). In a multivariate analysis of the study population, patients who started ART earlier (i.e., those with higher CD4 nadirs) more frequently achieved a normal ratio value. The persistence of a very low ratio (<0.3) predicted the risk of serious non-AIDS-related events or death, independent of CD4 count [20].

Another crucial aspect of health in PLWH is lipid metabolism. Significant concerns regarding metabolic profile alterations primarily stem from the literature indicating the onset of dyslipidemia following the switch from tenofovir disoproxil fumarate (TDF) to TAF [21,22,23,24]. Martini et al., investigated the possible metabolic role of TAF in antiretroviral-naïve HIV-infected patients comparing it with ABC/lamivudine backbone. They observed the development of hypercholesterolemia in the TAF group, that was not found in the no-TAF group. However, there were no significant differences regarding triglycerides, LDL, and the cardiovascular risk index. The role of this hypercholesterolemia was not clarified, and the only factor independently associated with hypercholesterolemia was the higher age, such as in the non-HIV population [24].

As expected, we observed a significant increase in total cholesterol, LDL, and BMI in the treatment-naïve population.

In the treatment-experienced patients, we found an inverse trend with a significant reduction in total cholesterol and triglycerides without changes in BMI. It should be noted that the analysis remained unchanged when excluding subjects with BMI < 18.5, i.e., considering only normal or over-weight subjects and excluding underweight ones (data not shown).

These data are not far from what emerged from the literature, which observed a significant increase in BMI and a relative reduction in triglycerides at 48 weeks [5]. Furthermore, another study demonstrated that after the switch to BIC-STR, patients with the worst lipid profiles at baseline had a significant improvement and patients switching away from PIs also showed triglyceride improvements [25].

Similar results were also highlighted in the BICTEL cohort, which shows a significant reduction in the total cholesterol/HDL ratio and the LDL value with a significant increase in BMI in regimens [16].

Therefore, according to our data and the literature data, the switch to BIC-STR should not be considered disadvantageous in metabolic terms but, on the contrary, could have favorable long-term effects, which are to be clarified in subsequent long-term studies. 

Surely, a great advantage of our study is that these data emerge from real clinical practice and are an expression of the use of the therapy in the field, therefore deeming them more representative than clinical studies designed to evaluate the efficacy of a drug.

However, our study has some limitations: the study is retrospective, and the sample is small and not homogeneous due to the differences between the number of experienced and naïve patients and age differences. Another limit is the “arbitrary” threshold selected as immunological recovery (CD4 > 500 cells/mm^3^) that does not consider that a CD4 count increasing, albeit good, does not reach the threshold. However, we considered this limit acceptable because only nine patients showed a CD4 increase of 50% in 1.5 years without reaching the value of 500 cells/mm^3^.

Furthermore, it would be important to evaluate the increase in body weight not only through BMI but also with impedancemetry. Finally, in light of the ageing of the population and the difference between biological age and chronological age, it would be important to evaluate direct and indirect markers of inflammation, in addition to the CD4/CD8 ratio: PCSK9, Lp(a), IL-6, ferritin, C reactive protein, evaluating any differences in the course of therapy with BIC-STR, especially in the older population.

## 5. Conclusions

While further and more extensive studies are certainly needed, this study has provided valuable insights, demonstrating the effectiveness of the BIC-STR regimen among treatment-naive and experienced patients, including those aged over 60 years old. Treatment discontinuation for adverse events was observed in a low number of cases.

This therapy demonstrated enhancements in virological control and immunological recovery, alongside beneficial metabolic changes post-switch. Consequently, it presents as a viable option for therapy transition and simplification, including as an initial ART in the older population.

## Figures and Tables

**Table 1:** Demographic characteristics of the population. Comparison of patients with follow-up more than six months and still in therapy and patients who discontinued BIC-STR.

	Patients Who Continued the Treatment, N 270	Patient Who Discontinued the Treatment, N 44	*P*
Age, median (IQR)	54.4 (44.6–61.2)	52.5 (42.1–60.4)	0.27
Male, N (%)	221 (81.9)	34 (77.3)	0.53
Italians, N (%)	206 (76.3)	35 (79.5)	0.84
CDC C, N (%)	74 (27.4)	9 (20.5)	0.36
Comorbidities > 2, N (%)	163 (60.1)	27 (61.3)	0.36
Hypertension, N (%)	59 (21.9)	7 (15.9)	0.43
CAD, N (%)	11 (4,7)	3 (6.8)	0.43
Stroke history, N (%)	8 (3.0)	1 (2.3)	0.99
Dyslipidaemia, N (%)	70 (25.9)	9 (20.5)	0.57
DM2, N (%)	28 (10.4)	3 (6.8)	0.59
Metabolic syndrome, N (%)	18 (6.7)	2 (4.5)	0.99
Neoplasia, N (%)	14 (5.2)	5 (11.4)	0.16
CKD, N (%)	8 (3.0)	1 (0.3)	0.99
Chronic liver disease, N (%)	40 (14.8)	11 (25)	0.07
Obesity, N (%)	29 (10.7)	3 (6.8)	0.59
Latent tuberculosis, N (%)	13 (4.8)	0 (0)	0.22
COPD, N (%)	13 (4.8)	2 (4.5)	0.99
BMI, median (IQR)	25.7 (23.2–28.4)	24.5 (22.5–27.1)	0.07
HCV ab positive, N (%)	51 (18.9)	15 (34.1)	0.03
HCV RNA detectable, N (%)	4 (1.5)	1 (2.3)	0.53
HBV infection, N (%)	93 (34.4)	9 (20.5)	0.08
Nadir CD4 count cell/mm^3^, median (IQR)	190 (50–348)	306.5 (207.5–488.0)	0.01
HIV-RNA Zenith cp/mL, median (IQR)	163,000 (52,500–642,000)	100,000 (25,000–311,000)	0.20
Previous ART (last before Biktarvy), N 242:			
INI, N (%)	187 (77.3)	29 (65.9)	0.73
NNRTI, N (%)	21 (8.7)	2 (4.5)	0.75
PI, N (%)	36 (14.9)	4 (9.1)	0.63
TAF backbone, N (%)	167 (69.0)	25 (56.8)	0.15
ABC backbone, N (%)	31 (12.8)	3 (6.8)	0.44
Dual-th, N (%)	15 (6.1)	2 (4.5)	0.99
Months on ART pre-switch, median, (IQR)	120 (48–180)	108 (48–198)	0.89
Availability of drug-resistance test, N (%)	183 (67.8)	23 (52.3)	0.03
Drug resistance (≥1), N (%)	58 (31.7)	6 (26.1)	0.64
Medical examination N/year, average (SD)	2.8 (1.4)	3.4 (2.4)	0.43

CDC, Centers for Disease Control and Prevention; ART, anti-retroviral therapy; INIs, integrase inhibitors; NNRTIs, non-nucleoside reverse transcriptase inhibitors; PIs, protease inhibitors; TAF, tenofovir alafenamide; ABC, abacavir; Dual-th, dual-therapy; CAD, chronic coronary artery disease; DMII, diabetes mellitus type II; CKD, chronic kidney disease; COPD, chronic obstructive pulmonary disease; BMI, body mass index; SD, standard deviation; IQR, interquartile range.

**Table 2:** Immuno-virological and metabolic variation after switch to BIC-STR in ART-experienced patients (N = 242).

	Pre-Switch Values	Last Values	*p*-Value
**CD4 count cell** **s** **/mm^3^, median (IQR)**	569.0 (388.0–823.0)	646.5 (445.3–882.5)	<0.0001
**CD4 %, median (IQR)**	31.0 (23.0–39.1)	33.0 (23.0–41.4)	<0.0001
**CD4/CD8 ratio, median (IQR)**	0.8 (0.5–1.1)	0.9 (0.4–1.3)	<0.0001
**HIV-RNA**			
**<50 cp/mL, N (%)**	197 (81.4)	228 (94.2)	<0.0001
**<20 cp/mL, N (%)**	166 (68.6)	203 (83.9)	<0.0001
**Undetectable (%)**	121 (50.0)	160 (66.1)	0.0003
**HIV-RNA median (IQR)**	nd (nd–27.5)	nd (nd–<20)	<0.0001
**Total cholesterol, median (IQR)**	198.0 (177.8–233.3)	195.5 (167.0–220.0)	0.0011
**HDL, median (IQR)**	49.0 (40.0–58.0)	50.0 (41.0–59.0)	0.96
**LDL, median (IQR)**	121.9 (97.5–148.7)	120.8 (95.3–144.8)	0.618
**Triglycerides, median (IQR)**	118.0 (85.8–180.0)	104.0 (74.3–154.8)	<0.0001
**BMI, median (IQR)**	25.7 (23.5–28.5)	25.8 (23.1–28.7)	0.4097

SD, standard deviation; IQR, interquartile range; BMI, body mass index; cp, copies; nd, not detectable.

**Table 3:** Immuno-virological and metabolic variation after BIC-STR introduction in ART naïve patients (N = 28). Median follow-up 1.8 years (IQR 1.3–2.5).

	Basal Values	Last Values	*p*-Value
**CD4 count cells/mm^3^, median (IQR)**	249.0 (61.5–425.5)	514.0 (217.5–697.3)	<0.0001
**CD4 %, median (IQR)**	13.3 (9.2–27.2)	24.5 (19.0–39.0)	<0.0001
**CD4/CD8 ratio, median (IQR)**	0.2 (0.1–0.6)	0.55 (0.4–0.9)	<0.0001
**HIV-RNA**			
**<50 cp/mL, N (%)**	0 (0)	26 (92.9)	
**<20 cp/mL, N (%)**	0 (0)	25 (89.3)	
**Undetectable, N (%)**	0 (0)	19 (67.9)	
**HIV-RNA cp/mL, median (IQR)**	387,000.0 (31,675–1,041,000)	nd (nd–<20)	<0.0001
**Total Cholesterol, median (IQR)**	179.0 (151.0–205.0)	195.5 (183.0–226.8)	0.0241
**HDL, median (IQR)**	42.0 (36.0–48.0)	46.0 (41.0–50.0)	0.0521
**LDL, median (IQR)**	105.4 (86.6–134.8)	125.6 (104.1–144.8)	0.0285
**Triglycerides, median (IQR)**	120.0 (82.0–148.0)	89.5 (69.8–161.5)	0.8367
**BMI, median (IQR)**	23.0 (22.1–24.8)	24.7 (23.5–27.2)	0.0156

SD, standard deviation; IQR, interquartile range; BMI, body mass index; cp, copies.

**Table 4:** Immuno-virological and metabolic variation after switch to BIC-STR in ART-experienced patients aged over 60 years old (N = 81).

	Pre-Switch Values	Last Values	*p*-Value
**CD4 count, cells/mm^3^, median (IQR)**	528.0 (339.0–794.0)	581.5 (345.3–864.8)	<0.0001
**CD4 %, median (IQR)**	29.5 (20.8–37.7)	30.9 (23.6–41.3)	<0.0001
**CD4/CD8 ratio, mean (SD)**	0.9 (0.8)	1.0 (0.8)	<0.0001
**HIV-RNA**			
**<50 cp/mL, N (%)**	69 (85.2)	75 (92.6)	0.021
**<20 cp/mL, N (%)**	58 (71.6)	69 (85.2)	0.036
**Undetectable (%)**	41 (50.6)	54 (66.7)	0.038
**HIV-RNA median (IQR)**	nd (nd–23.0)	nd (nd–<20)	0.0032
**Total cholesterol, median (IQR)**	196.0 (169.5–223.0)	188.0 (160.5–217.3)	0.0321
**HDL, median (IQR)**	47.0 (38.0–57.0)	46 (39.0–58.5)	0.6510
**LDL, median (IQR)**	119.0 (90.5–141.3)	113.5 (88.0–145.0)	0.6367
**Triglycerides, median (IQR)**	121.0 (92.3–187.8)	114.0 (80.5–154.3)	0.0013
**BMI, median (IQR)**	25.8 (23.9–28.2)	25.7 (23.8–28.0)	0.1657

SD, standard deviation; IQR, interquartile range; BMI, body mass index; cp, copies; nd, not detectable.

## Data Availability

The datasets analyzed during the current study are available from the corresponding author on reasonable request.

## Supplementary Materials

The following supporting information can be downloaded at: https://www.mdpi.com/article/10.3390/idr15060069/s1, Figure S1: Causes of bictegravir/tenofovir alafenamide/emtricitabine interruption in 44 cases, expressed as percentage of total. Dual-th, dual therapy.; Table S1: Multiple logistic regression, variables that influence the reach of target undetected viermia. The model correctness was verified with Hosmer-Lemeshow test (value 5.6, *p* = 0.6898). Table S2: Multiple logistic regression, variables that influence a good immunological recovery, defined as CD4+ T cell count > 500 cells/mm3 in overall population. The model correctness was verified with Hosmer-Lemeshow test (value 8.6, *p* = 0.3802). Table S3: Multiple logistic regression, variables that influence a good immunological recovery, defined as CD4 count > 500 cell/mm3 patients aged over 60 years old. The model correctness was verified with Hosmer-Lemeshow test (value 8.7, *p* = 0.3668).
