# Supplementary material for: Bictegravir/Tenofovir Alafenamide/Emtricitabine: A Real-Life Experience in People Living with HIV (PLWH)
Source: Infect Dis Rep. 2023 Dec 11;15(6):766–77. doi: 10.3390/idr15060069 (PMC10742537; doi:10.3390/idr15060069)
Supplement: Supplementary file 1 [file idr-15-00069-s001.zip › idr-2620345-supplementary.pdf]

Supplementary Table S1: Multiple logistic regression, variables that influence the reach of target undetected viermia. The model correctness was verified with Hosmer-Lemeshow test (value 5.6, p=0.6898).

Variables	Odds ratio	95% CI
**Zenith viremia cp/ml**	1.000	1.000 to 1.000
**CD4 nadir count, cell/mm^3**	1.000	0.9988 to 1.002
**Years on BIC-STR**	1.643	1.038 to 2.660
**Age**	0.9837	0.9511 to 1.016

CI, confidence interval.

Supplementary Table S2: Multiple logistic regression, variables that influence a good immunological recovery, defined as CD4+ T cell count > 500 cells/mm^3 in overall population. The model correctness was verified with Hosmer-Lemeshow test (value 8.6, p=0.3802).

Variables	Odds ratio	95% CI
**Previous TAF-based regimen**	2.642	1.235 to 5.787
**Zenith viremia, cp/ml**	1.000	1.000 to 1.000
**CD4 nadir, cells/mmc**	1.006	1.004 to 1.009
**OI**	0.7488	0.3035 to 1.838
**HCV co-infection**	0.6101	0.2232 to 1.649
**Years on BIC-STR**	0.9008	0.5911 to 1.364
**Age**	0.9872	0.9562 to 1.019

TAF, Tenofovir Alafenamide; OI, previous opportunistic infection; CI, confidence interval;

Supplementary Table S3: Multiple logistic regression, variables that influence a good immunological recovery, defined as CD4 count > 500 cell/mm^3 patients aged over 60 years old. The model correctness was verified with Hosmer-Lemeshow test (value 8.7, p=0.3668).

**Variables**	**Odds ratio**	**95% CI**
**Previous TAF-based regimen**	1.365	0.3032 to 6.080
**Zenith viremia, cp/ml**	1.000	1.000 to 1.000
**CD4 nadir count, cells/mm^3**	1.003	0.9982 to 1.008
**Age**	0.7879	0.6372 to 0.9386
**OI**	0.1378	0.01913 to 0.7464

TAF, Tenofovir Alafenamide; CI, confidence interval; OI, previous opportunistic infection

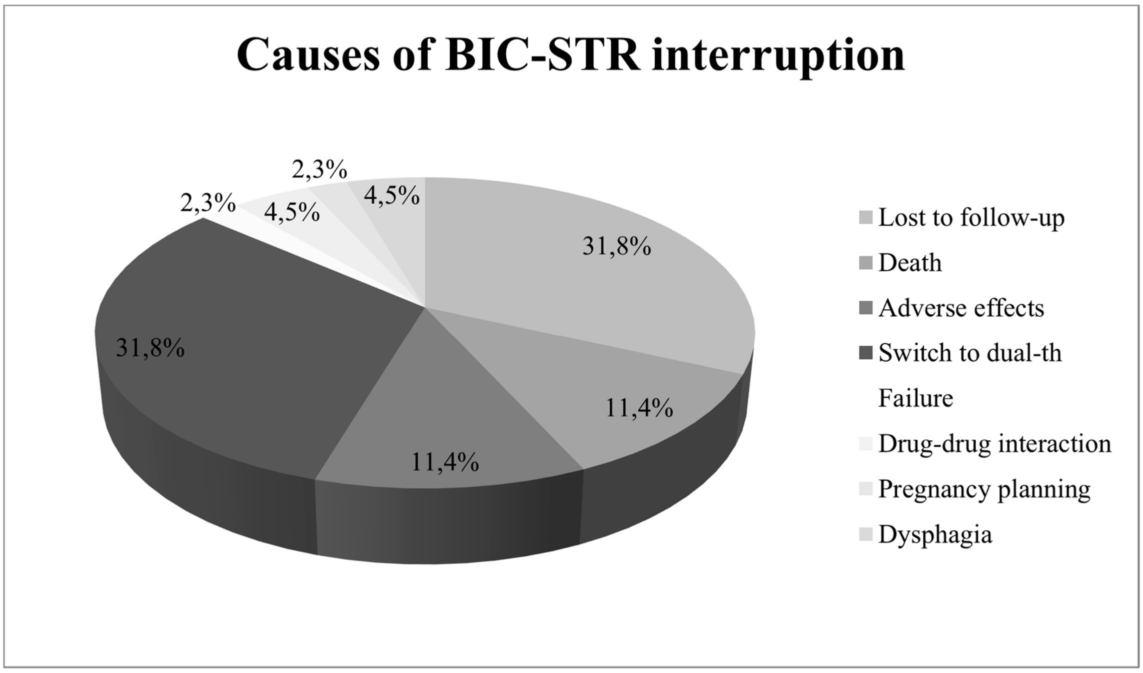

Supplementary Figure S1: Causes of bictegravir/tenofovir alafenamide/emtricitabine interruption in 44 cases, expressed as percentage of total. *Dual-th, dual therapy.*
